# Impacts of nitrogen fertilization rate on the root yield, starch yield and starch physicochemical properties of the sweet potato cultivar Jishu 25

**DOI:** 10.1371/journal.pone.0221351

**Published:** 2019-08-22

**Authors:** Wenxue Duan, Haiyan Zhang, Beitao Xie, Baoqing Wang, Liming Zhang

**Affiliations:** 1 Crop Research Institute, Shandong Academy of Agricultural Sciences, Jinan, Shandong, China; 2 Scientific Observation and Experimental Station of Tuber and Root Crops in Huang-Huai-Hai Region, Ministry of Agriculture and Rural Affairs, Jinan, Shandong, China; 3 Shandong Academy of Agricultural Sciences, Jinan, Shandong, China; 4 College of Life Sciences, Shandong Normal University, Jinan, Shandong, China; North Eastern Regional Institute of Science and Technology, INDIA

## Abstract

In recent years, the sweet potato cultivar Jishu 25 has exhibited good characteristics for starch processing in northern China. The storage root dry matter yields of this cultivar can exceed one ton per mu (1/15 of a hectare) at nitrogen (N) rates of 60–90 kg ha^-1^ based on soil nutrient content. However, the effect of N fertilizer on the physicochemical properties of starches isolated from this cultivar has not been reported. In order to evaluate these effects, three different N rates, 0 (control, N0), 75 (N1), and 150 kg ha^-1^ (N2), were selected for a field experiment in 2017. The results showed that N1 exhibited the highest storage root yield and starch yield. Compared to the control group, N fertilizer significantly increased the total starch content while no significant difference was found in these between the N1 and N2 groups. The amylose (AM) content was highest in the N2 group and lowest in the N0 group. In addition, N fertilizer exhibited no significant effects on the values of [D(*v*, 0.9)], D [4, 3] and D [3, 2]. Compared to the control group, N1 demonstrated significantly higher setback viscosity (SV), while N2 showed significantly higher peak viscosity (PV), cold paste viscosity (CPV) and SV. However, there were no significant differences in the hot paste viscosity (HPV), peak time and pasting temperature between the N1 and N2 groups. For the thermal properties of starch, there were no significant differences in peak temperature (T_p_), conclusion temperature (T_c_) or gelatinization enthalpy (*Δ*H) between the N1 and N2 groups. Overall, for the starch samples of cultivar Jishu 25, N fertilizer exerts significant effects on the starch content, AM content and viscosity properties but little effect on the particle size distribution and *ΔH*. 75 kg N ha^-1^ can easily lead to substantial planting benefits from the high storage root yield, dry matter yield and total starch content of this cultivar.

## Introduction

Sweet potato has been used globally for vegetable and staple food production and as a raw material for industrial processing [1]. Sweet potatoes are commonly used in the food industry for the production of breads, cakes, chips, and noodles, as well as for transformation into biomaterials such as bioethanol and modified starches [2–5]. Starch is a main component in tuberous roots, accounting for approximately 50–80% of their dry weight [5]. This is one of the primary reasons that starch is an abundantly available and inexpensive agricultural product [2, 4]. The dry matter content of tuberous roots is mainly attributed to starch accumulation, and thus, the amount of starch accumulation indicates the yield of dried tuberous roots. Moreover, starch content, amylose (AM) content, and particle size distribution are closely related to the characteristics of starch in tuberous roots [6–8]. Hence, a better understanding of the relationship between the functional and structural properties of sweet potato starch is crucial for the optimization of its food and industrial applications [8].

In China, the sweet potato harvest area is 3281.52 thousand hectares, and the total production was 70.57 million tons in 2016, ranking first in the world [9]. The proportion of planting areas of starch-producing sweet potato cultivars is approximately 40–50% in China [10]. Although numerous sweet potato cultivars are produced by the Chinese starch industry, only a few studies have reported on their specific physicochemical properties [3, 11–13]. The cultivar Jishu 25 was approved by the National Crop Variety Approval Committee of China in 2016. In 2018, the exclusive right of implementation for this high-starch cultivar was successfully transferred at the price of 1 million RMB over five years to Sishui Lifeng Food Co., Ltd., the largest sweet potato starch enterprise in China, with an annual starch processing capacity of 120 thousand tons per year. However, there are limited reports on the effects of cultivation measures on the physicochemical properties of starch in this cultivar.

Nitrogen (N) is an abundant nutrient element necessary for the growth and development of sweet potato. Appropriate N fertilizer input is one of the effective measures that increases the storage root yield of sweet potato. Phillips et al. [14] showed that optimum N application rates varied annually under different precipitation and 28–56 kg N ha^-1^ was required for maximum root yield of 'Beauregard' sweet potato in Virginia. In subsistence sweet potato production systems of humid tropical Papua New Guinea, mineral N fertilizer 25 kg N ha^-1^ plus poultry manure 25 kg N ha^-1^ produced significantly greater total tuber yields than in controls [15]. However, excessive usage of N fertilizer application can cause vine overgrowth and lower yield [16]. Additionally, N supply produced significant effects on the starch content of tuberous roots in sweet potato [17]. Also, N application could affect physicochemical properties of starch samples isolated from sweet potato and it may increase the peak viscosity (PV), hot paste viscosity (HPV), cool paste viscosity (CPV) and setback viscosity (SV) [18]. However, according to Noda et al. [19], starches properties from some sweet potato cultivars were not altered by increasing the N fertilizer. Reasonable adjustment of N fertilizer to maximize root yield and reduce adverse effects on quality has become an urgent matter in sweet potato production. In recent years, the Chinese agriculture research system has enabled researchers to screen suitable sweet potato cultivars with high starch content and develop cultivation techniques for increasing the dry matter yield of tuberous roots to over one ton per mu (1/15 of a hectare) for these cultivars. Notably, the application rate of N is approximately 60–90 kg ha^-1^ based on the soil nutrient content for the cultivar Jishu 25. To the best of our knowledge, the effect of N fertilizer on the physicochemical properties of starches isolated from the Jishu 25 cultivar has not been reported. Therefore, this study aimed to investigate these effects at different N rates, and the results may provide a scientific description of the impacts of N fertilizer on the large-scale production of high-quality sweet potato cultivars.

## Materials and methods

### Experimental design

Field experiments were conducted on sandy loam soils in Liujia Village (36°8′ N, 117°6′ E), Zouping, Binzhou, Shandong Province, China from May to October 2017. The mean annual rainfall and temperature for the area were 639.9 mm and 13.6°C, respectively, with 60–70% of rainfall during the summer months of June, July, and August. Before sowing, the nutrient contents of the top 20 cm soil layer were 11.6 g kg^-1^ organic matter, 0.9 g kg^-1^ total N, 52.5 mg kg^-1^ alkali-hydrolyzable N, 24.2 mg kg^-1^ available phosphate and 89.7 mg kg^-1^ available potassium. No specific permissions were required to work at the experimental site.

A starch-producing long-vine cultivar (Jishu 25) was selected for this study. Recently, the Chinese agricultural research system has recently screened suitable sweet potato cultivars with high starch content and has developed cultivation techniques for increasing the dry matter yield of the tuberous roots to over 1 ton per mu. For the cultivar Jishu 25, the application rate of N is approximately 60–90 kg ha^−1^ based on soil nutrient content. Therefore, three application rates of N have been selected on the basis of the nutrient contents of the experimental field. These rates are as follows: N0 (control), N1 (75 kg ha^−1^), and N2 (150 kg ha^−1^). The fertilizers used included urea (containing 46.4% N), calcium triple superphosphate (containing 46% P_2_O_5_) and potassium sulfate (containing 50% K_2_O). Constant amounts of P_2_O_5_ (75 kg ha^-1^) and K_2_O (150 kg ha^-1^) were combined with the varying concentrations of N as a base fertilizer. All cultivars were planted on May 15, 2017, with an in-row spacing of 80 cm × 24 cm (row width × plant space) and were harvested on October 15, 2017. All experiments were replicated three times in 80 m^2^ plots under a randomized complete block design. Other aspects of cultivation were similar to those used in normal fields. We obtained the relevant permission from the corresponding institute (Crop Research Institute, Shandong Academy of Agricultural Sciences, China) to plant our materials in the field. We confirm that the field studies did not involve endangered or protected species.

### Starch content determination

Powdered tuberous roots were baked for 2 days at 80°C until they reached a constant dry weight (DW). The 30-mg dried samples were dissolved in 80% ethanol (0.7 ml), mixed thoroughly by vortexing and then incubated for 2 hours at 70°C. After inverting several times, the mixture was centrifuged for 10 minutes at 12000 *g*. The resulting pellets were collected and washed 3 times. After washing, the mixture was boiled and then enzymolysis was performed via thermostable α-amylase followed by amyloglucosidase. Glucose concentrations were measured by the colorimetric method at 510 nm using glucose oxidase-peroxidase-aminoantipyrine against a reagent blank. The total content of starch was expressed on a DW basis [20–21].

### Starch isolation

Starches were isolated from sweet potato roots according to the method of Zhu et al. [12]. Briefly, the tuberous roots were cleaned, peeled and cut into smaller pieces, and then macerated with a small amount of water. After blending, the resultant slurry was washed and sieved several times to eliminate impurities. Subsequently, the purified starches were dehydrated in an oven (Shanghai Yiheng Technology Ltd., Shanghai, China) at 40°C for 24 hours. After drying, the starches were ground into fine powder and stored in a sealed container awaiting further analyses.

### AM content determination

The concentrations of AM in starch samples were measured by a colorimetric method based on amylose-iodine complex formation as described previously [22]. Briefly, 100 mg of starch sample was placed in a flask and mixed with 9 mL of 1 M NaOH and 1 mL of 95% ethanol. Subsequently, the mixture was heated for 15 minutes in a water bath. After cooling to room temperature, the volume was adjusted to 100 mL with ddH_2_O (double-distilled water). After vigorously mixing, 5 mL of the solution was transferred into a new flask with 1 mL of acetic acid (1 M), 2 mL iodine solution and 50 mL of ddH_2_O. Afterwards, the final volume was adjusted to 100 mL with ddH_2_O, followed by vigorous mixing and incubation for 20 minutes. A spectrometer was used to measure the absorbance at 620 nm. To establish a standard curve, AM (A0512, Sigma-Aldrich, USA) and amylopectin (AP; A-10118, Sigma-Aldrich, USA) were used as reference standards. The concentrations of AP were calculated as follows: (100—AM %).

### Measurement of pasting properties

The pasting properties of starch samples were determined with a Rapid Visco-Analyzer (RVA-4, Newport Scientific Pty. Ltd., Warriewood, Australia). Briefly, a 25 g powdered sample was weighed and dissolved in distilled water to a final concentration of 10%. The test profile was programmed by following the procedures of RVA Standard Profile 1 [21]. Pasting parameters such as pasting temperature, PV, HPV, and CPV were determined, and the breakdown viscosity (BV = PV-HPV) and SV (SV = CPV-HPV) were subsequently calculated [7].

### Particle size distribution of starch

The particle size distributions of starch samples were measured by a laser diffraction particle size analysis instrument (Master-size 3000; Malvern Instruments Ltd., Worcestershire, UK) with a water-based dispersing medium. Briefly, starch was added to the reservoir, and the refractive indices of 1.33 and 1.50 were used for water and starch, respectively [21]. The parameters of particle size distribution were derived through equipment software as follows: 10th [D(*v*, 0.1)], 50th, median [D(*v*, 0.5)] and 90th [D(*v*, 0.9)] percentiles, as well as surface weighted mean diameter D[3, 2] and volume weighted mean diameter D[4, 3] [6, 23].

### Thermal properties of starch

The thermal properties of starch samples were determined using a differential scanning calorimeter (DSC; Q2000, TA instruments, New Castle, DE, USA). The suspension of starch and distilled water (1:3, w/w) was sealed in an aluminum pan and equilibrated for 24 hours at room temperature prior to DSC analysis. Meanwhile, an unfilled aluminum pan was used as the reference control. Subsequently, the samples were heated to 30–95°C at a rate of 10°C per minute. Thermal parameters such as onset temperature (T_o_), peak temperature (T_p_), conclusion temperature (T_c_) and gelatinization enthalpy (*ΔH*) were determined [7].

### Statistical analysis

All statistical analyses were conducted using SPSS version 17.0 for Windows (SPSS, Chicago, Illinois, USA). The differences between means were compared by ANOVA. Duncan's multiple range test was used to estimate the differences in means between treatment groups. P-values of less than 0.05 were regarded as statistically significant.

## Results

### Root and starch yields

The total number of tuberous roots was highest in the N0 group and lowest in the N2 group (Table 1). The weight of tuberous roots and yield of fresh storage roots in the N1 group were significantly higher than in the N0 and N2 groups. Among them, the N0 group exhibited the lowest tuberous root weight and fresh storage root yield. The dry matter and total starch contents of plants in the N1 and N2 groups were not significantly different but were significantly higher than those in the N0 group. The changes in starch yield at different N rates were relatively similar to those for fresh storage root yield. These results suggest that the N application rate of 75 kg ha^-1^ yielded the greatest amounts of starch and fresh storage roots.

**Table 1:** Fresh storage root yield, dry matter ratio and starch yield under different treatments.

Treatment	Number of tuberous roots per plant	Tuberous root weight per plant (g)	Fresh storage root yield (kg ha^-1^)	Dry matter ratio (%)	Total starch content (%)	Starch yield (kg ha^-1^)
**N0**	4.23a	630.80c	31536.70c	33.16b	65.65b	6865.39c
**N1**	3.93b	826.26a	41308.71a	38.12a	70.58a	11114.15a
**N2**	2.96c	725.39b	36265.72b	39.53a	72.61a	10409.25b

The values in the same column with different letters differ significantly (p < 0.05).

### AM content, AP content and AP/AM ratio

The AM content was highest in the N0 group and lowest in the N2 group (Table 2). Notably, the AP contents of plants in the N2 group were significantly increased compared to those in the N0 group, and no significant difference in AP content was found between the N1 and N2 groups. Moreover, the AP/AM ratio was highest in the N2 group and lowest in the N0 group. These results suggest that 75 kg N ha^-1^ reduces AM content and increases the AP/AM ratio. Additionally, elevating the N rate to 150 kg ha^-1^ further affects the AM content and the AP/AM ratio.

**Table 2:** The total starch content, amylose content and amylopectin content under different treatments.

Treatment	Amylose content (%)	Amylopectin content (%)	Amylopectin/Amylose
**N0**	28.39a	70.61b	2.49c
**N1**	25.73b	74.27ab	2.89b
**N2**	24.03c	75.97a	3.16a

The values in the same column with different letters differ significantly (p < 0.05).

### Particle size distribution

**Fig 1:**
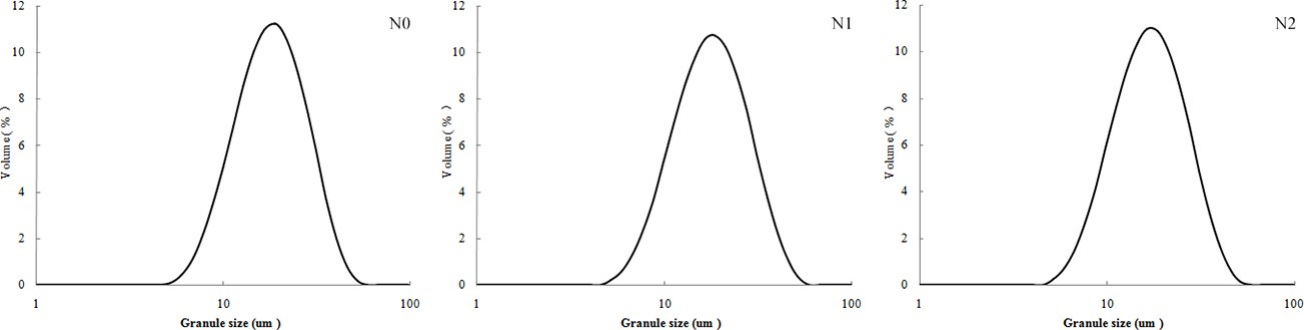
Comparisons of the size distribution of starch granules under different treatments.

The starch granules of cultivar Jishu 25 displayed a unimodal size distribution and ranged from 5 to 60 μm (Fig 1), with most from 10 to 34 μm (Table 3). [D(v, 0.1)] and [D(v, 0.5)] values in the N0 group were significantly higher than in the N2 group. However, no significant differences were found in those between the N1 and N2 groups. Additionally, [D(*v*, 0.9)] values were not significantly different among the three groups. Likewise, D [4, 3] and D [3, 2] values exhibited similar trends as those of [D(*v*, 0.9)]. These results indicate that N fertilizer exerts minor effects on the particle size distribution of starch granules.

**Table 3:** The particle size parameters under different treatments.

Treatment	[D(*v*, 0.1)]	[D(*v*, 0.5)]	[D(*v*, 0.9)]	D [4, 3]	D [3, 2]
**N0**	10.68a	19.06a	32.85a	19.32a	16.05a
**N1**	10.33ab	18.87ab	33.66a	19.18a	15.80a
**N2**	10.12b	17.96b	31.38a	18.34a	15.28a

[D(v, 0.1)], [D(v, 0.5)], [D(v, 0.9)] indicate 10th, 50th (median) and 90th percentiles, respectively. D [4,3], volume-weighted mean diameter; D [3,2], surface-weighted mean diameter. The values in the same column with different letters differ significantly (p < 0.05).

### Pasting properties

**Fig 2:**
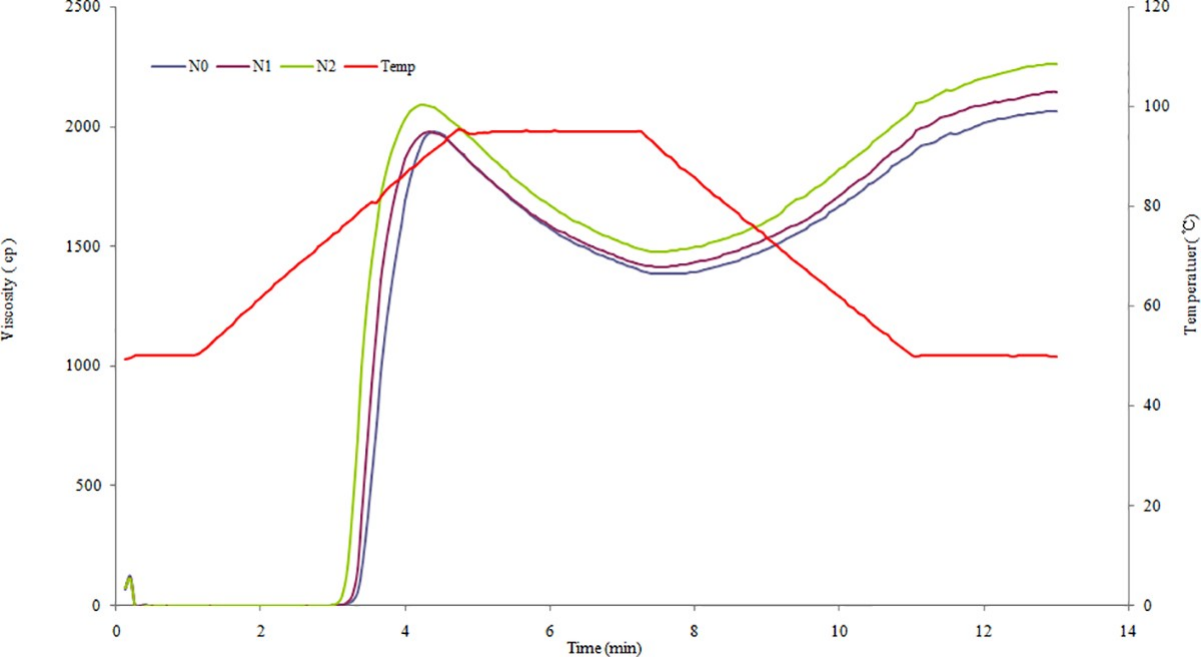
Rapid Visco-Analyser pasting profiles of starches from sweet potato with different treatments.

The pasting profiles of starches were determined using RVA (Fig 2), and the results for pasting parameters are shown in Table 4. The PV and CPV in the N2 group were significantly higher than those in the N0 and N1 groups. There were no significant differences in those between the N0 and N1 groups. The HPV in the N2 group was significantly higher than in the N0 group, but no significant difference was found when comparing the N2 and N1 groups. The N2 group exhibited significantly higher BV values than in the N1 group, but no significant difference was found between them in the N0 and N1 groups. The SV in the N0 group was the lowest, while in the N2 group, it was the highest. There was no significant difference in the peak time among the three groups. The pasting temperature in the N2 group was significantly lower than in the N0 group, but no significant difference was found in the N1 and N2 groups. Compared to the control group, 75 kg N ha^-1^ increased the SV level, while 150 kg N ha^-1^ elevated the levels of PV, HPV, CPV and SV and reduced the pasting temperature. The two N rates of 75 and 150 kg ha^-1^ had no significant effects on HPV, peak time or pasting temperature.

**Table 4:** Viscosity properties of starches under different treatments.

Treatment	Peak viscosity (cP)	Hot paste viscosity (cP)	Breakdown (cP)	Cold past viscosity (cP)	Setback (cP)	Peak time (min)	Pasting temperature (°C)
**N0**	1978b	1384b	594ab	2062b	678c	4.40a	77.55a
**N1**	1977b	1412ab	565b	2146b	734b	4.33a	76.75ab
**N2**	2092a	1475a	617a	2261a	786a	4.27a	75.10b

The values in the same column with different letters differ significantly (p < 0.05).

### Thermal properties

**Fig 3:**
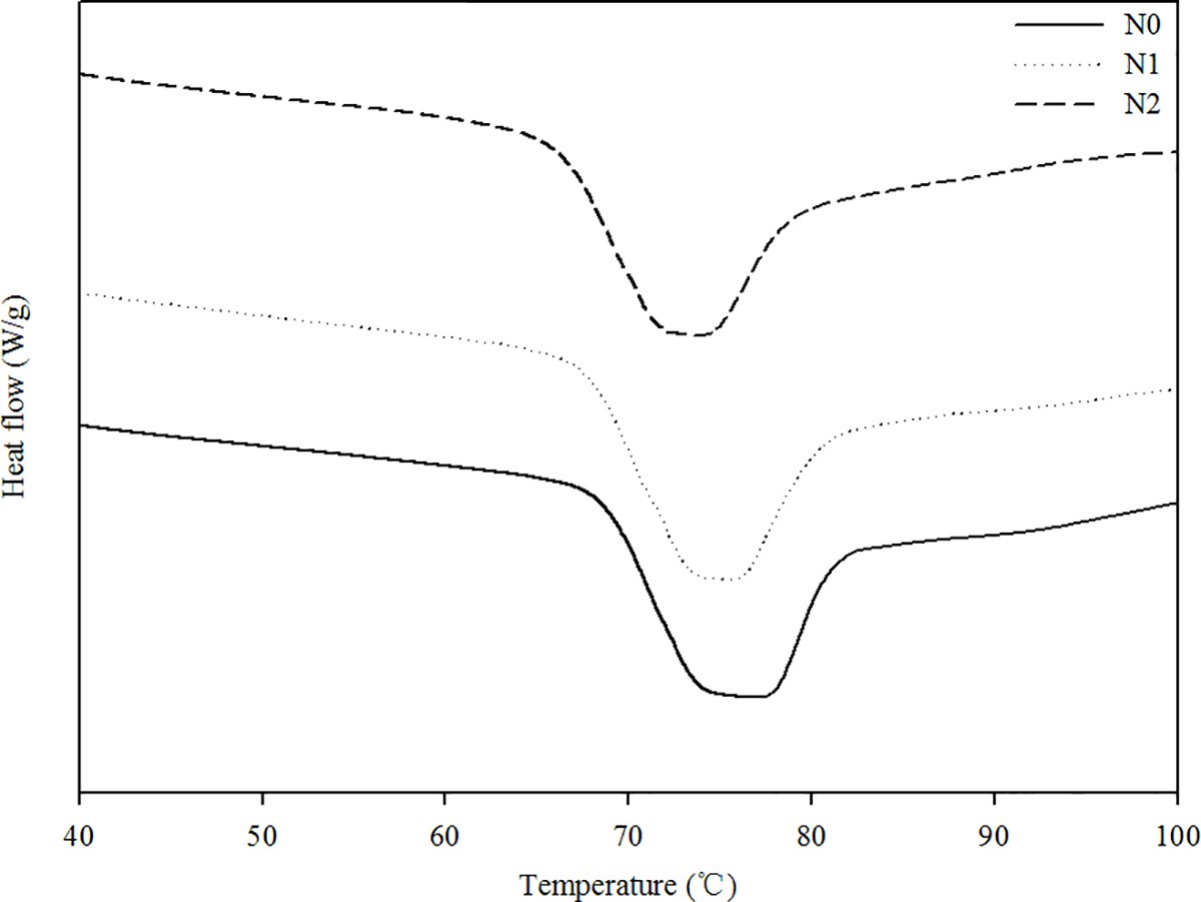
Differential scanning calorimeter thermograms of starches from different treatments.

The DSC curves under different N rates are shown in Fig 3, and the results for thermal parameters, such as T_o_, T_p_, T_c_ and *ΔH*, are presented in Table 5. The T_o_ value was highest in the N0 group and lowest in the N2 group. The T_p_ value in the N0 group was significantly increased compared to in the N2 group, and no significant difference was found between the N1 and N2 groups. Tc values in the N0 and N1 groups were significantly higher than in the N2 group, and there was no significant difference in the Tc value between the N0 and N1 groups. Additionally, *ΔH* values were not significantly different among the three N rates. These results suggest that N fertilizer decreases T_o_ values, and 150 kg N ha^-1^ further reduces the values of T_o_ and T_c_. However, N fertilizer exhibits no significant effect on *ΔH*.

**Table 5:** Thermal properties of starch gelatinization under different treatments as determined by differential scanning calorimeter (DSC).

Treatment	T_o_ (°C)	T_p_ (°C)	T_c_ (°C)	*Δ*H (J·g^-1^)
**N0**	68.90a	74.66a	80.92a	10.30a
**N1**	67.85b	73.75ab	80.25a	10.25a
**N2**	66.67c	72.05b	78.49b	9.81a

T_o_, T_p_, T_c_, *Δ*H indicate gelatinization parameters onset, peak, conclusion temperature, and gelatinization enthalpy, respectively. The values in the same column with different letters differ significantly (p < 0.05).

## Discussion

Starch yield is a parameter for determining the benefit of planting sweet potato. The use of high-starch cultivars by farmers needs to be encouraged by the processing industries, and farmers should be reawarded with appropriate performance incentives [8]. For sweet potato cultivar Rainha Branca, starch levels are reduced with increasing urea levels, with the lowest starch percentage at a urea level of 460 kg ha^-1^ [24]. However, for sweet potato cultivars Xushu 22 and Xushu 28, N application rates of 60–120 kg ha^-1^ may not greatly decrease the contents of starch and AM but a rate of 180 kg N ha^-1^ significantly reduces the storage root yields of both cultivars [18]. The appropriate amount of N application varies with different planting environments and cultivars. In this study, for cultivar Jishu 25, the starch content and total starch yields ranged from 65.65 to 72.61 g/100 g DW and 6865.39 to 11114.15 kg ha^-1^, respectively, under N application rates of 0–150 kg ha^-1^. A application rate of 75 kg N ha^-1^ produced significantly higher total starch content and lower AM content than those without N cultivation. Raising the N rate to 150 kg ha^-1^, produced no significant difference in total starch content but starch yield was significantly decreased because of the reduced storage root yield. Additionally, the highest dry matter accumulation in tuberous roots cultivated with 75 kg N ha^-1^ was 1049.79 kg per mu, which exceeded one ton per mu. Therefore, applying 75 kg N ha^-1^ easily achieves high planting benefits, as revealed by the high yields of storage roots, dry matter and total starch for cultivar Jishu 25.

Hoover [25] produced sweet potato starch with an average size of 19 μm, ranging from 2 to 42 mm. In another study, the highest mean granule size of sweet potato starch was 13.07 mm with a broad granule size range of 0.85–44.69 mm, whereas the lowest mean granule size was 8.10 mm with a narrow granule size range of 0.76–29.12 mm [13]. These divergent results may be attributed to the different cultivars, growing conditions and differences in plant physiology. In this study, the granule sizes of starch in Jishu 25 ranged from 4.03 to 58.88 mm, which was higher than in previous findings. According to Noda et al. [19], the average granule sizes of four different sweet potato cultivars may not respond to NPK fertilizer treatments. In this study, 75 kg N ha^-1^ exhibited no significant effects on the parameters of particle size, while 150 kg N ha^-1^ decreased both the 10th and 50th percentiles when compared to the particle size in plants without N application. The two rates of added N fertilizer revealed no significant difference in these parameters, suggesting that different N rates have little effect on the particle size distribution of cultivar Jishu 25.

The pasting properties, such as PV, BV, SV and peak time varies significantly among sweet potato starches [13]. Likewise, the pasting temperature of sweet potato starches varies greatly, from 50.0 to 86.6°C [12, 13, 26]. In this study, the viscosity properties of Jishu 25 starch, such as PV, BV, SV and CPV, varied among the three N rates, while the pasting temperature ranged from 75.10 to 77.55°C. For starch extracted from four sweet potato cultivars in Japan, higher NPK fertilization rates did not cause a significant change in the RVA-measured values [19]. However, for two cultivars of Xushu 22 and Xushu 28 in China, 120 kg N ha^-1^ significantly increased the levels of PV and CPV. Compared to 60 kg N ha^-1^, the excessive N rate of 240 kg ha^-1^ significantly increased the levels of PV, HPV and CPV for cultivar Xushu 22 [18]. In this study, 75 kg N ha^-1^ increased the SV level compared to non-N cultivation, and elevating the N rate to 150 kg ha^-1^ further increased the levels of PV, CPV and SV. This maybe related to the lower AM content of the starch samples caused by applying 150 kg N ha^-1^. Previous studies have shown that the *ΔH* of sweet potato starches ranges from 7.6 to 15.8 J/g [12, 27]. Furthermore, the different gelatinization behaviors might lead to the inconsistency in *ΔH* [7]. The increased NPK fertilization rates had no significant effects on gelatinization behaviors and thus little influence on DSC-measured gelatinization properties [19]. In this study, the *ΔH* of Jishu 25 starch ranged from 9.81 to 10.30 J/g, which falls within the defined range for this species. N fertilizer significantly decreased the AM content of the starch samples and affected some of the parameters of pasting property. However, 75 or 150 kg N ha^-1^ did not significantly affect *ΔH* values. This suggests that the *ΔH* is affected by many aspects of starch granule gelatinization and it is difficult to link *ΔH* to only one factor. Moreover, the N rate of 150 kg ha^-1^ with a lower transition temperature exhibited no significant changes in *ΔH* compared to the control with a higher transition temperature, which is incompatible with the results of Abegunde et al. [13]. This inconsistency can probably be attributed to cultivar differences and growing conditions.

Starches with different AM contents are of great interest due to their wide applicability and impacts on final product characteristics [28]. Starches with lower AM contents can be gelatinized easily and generate clearer pastes. The firmness of starch noodles is significantly associated with certain RVA pasting characteristics, and an RVA viscoamylograph can be used to determine the differences in pasting characteristics of sweet potato starch that relate to noodle quality [29]. However, the findings for the relationship between AM content and viscosity parameters are not consistent. Tester and Morrison [30] demonstrated that starch granular swelling is mainly a feature of AP and that AM appears to inhibit the swelling of starch granules. The levels of PV, HPV and CPV were negatively influenced by AM content [29]. Similar results were observed in other studies [6, 31–32]. In contrast, a positive association between AM content and the levels of PV, CPV and BV was found in another study [33]. Some other studies suggested a non-significant relationship between AM content and paste viscosities, even though the AM contents of starches are altered [13, 34]. In this study, the starches in the N2 group (150 kg N ha^-1^) with low AM content exhibited higher PV, CPV and SV levels and lower pasting temperature compared to those in the control group with high AM content. These results were consistent with data from only some previous studies [6, 32]. Genotype might be one of the main factors that resulted in these differences. On the other hand, differences in gelatinization characteristics might be attributed mainly to the molecular structural diversities of starch [31]. Our results showed that the AM content varied greatly between the N1 and N2 groups, while no significant changes were observed in HPV, peak time and pasting temperature. The underlying reasons may be related to the molecular structure of starch, such as the internal unit chain composition of AP and long AM molecular structure [6–7]. Additionally, sweet potato growth may be subjected to different cultivars, locations and processes to obtain the optimal starch quality parameters for specific industrial uses. Furthermore, improved starch characteristics for industrial purposes can be achieved by chemical and physical modifications [8]. In this study, N fertilizer is an effective measure for the regulation of crop yield and starch for cultivar Jishu 25. Considering the higher yields of dry matter and starch, lower N input and higher agricultural income, 75 kg ha^-1^ is the most cost-effective N application rate.

## Conclusion

Overall, N fertilizer exerts significant effects on the total starch content, AM content and viscosity properties (e.g., PV, CPV and SV) of the cultivar Jishu 25 but little effect on the *ΔH* and particle size distributions (e.g., 90th percentiles, surface-weighted mean diameter and volume-weighted mean diameter) of the starch samples. Notably, 75 or 150 kg N ha^-1^ had no significant difference on the mean diameter of starch particles, HPV, peak time, peak temperature and *ΔH* of the starch samples. Additionally, the 75 kg N ha^-1^ treatment resulted in the highest dry matter content in the storage roots and yielded the greatest amounts of starch and storage roots, indicating that it is a cost-effective N rate for cultivating Jishu 25.

## Supporting information

S1 File The relevant data in Tables 1–5 and Figs 1 and 2. (XLS)

S2 File The relevant data in Fig 3. (RAR)

## Abbreviations

AM: amylose
AP: amylopectin
BV: breakdown viscosity
CPV: cold paste viscosity
HPV: hot paste viscosity
ΔH: gelatinization enthalpy
N: nitrogen
PV: peak viscosity
SV: setback viscosity
T_c_: conclusion temperature
T_o_: onset temperature
T_p_: peak temperature
